# Rare Bi-focal Presentation of Avascular Necrosis of the Femoral Head: Successful Targeted Treatment as per the Diamond Concept and Review of the Literature

**DOI:** 10.7759/cureus.36423

**Published:** 2023-03-20

**Authors:** Sophia M Wakefield, Peter V Giannoudis

**Affiliations:** 1 Academic Department of Trauma & Orthopaedic Surgery, School of Medicine, University of Leeds, Leeds, GBR; 2 Trauma and Orthopaedics, National Institute for Health and Care Research (NIHR) Leeds Biomedical Research Centre, Chapel Allerton Hospital, Leeds, GBR

**Keywords:** avascular necrosis of the femoral head, avascular necrosis, diamond concept, decompression, femoral head, avascular necrosis (avn), bi-focal

## Abstract

Avascular necrosis of the femoral head (AVNFH) is relatively infrequent, but if undiagnosed or untreated, it may result in significant functional disability, and due to severe ongoing pain, a total hip replacement (THR) may be necessitated. Most cases are associated with trauma, but a number of established risk factors exist. Diagnosis can be challenging but relies on clinical history, physical examination, and radiology. X-ray and MRI are used to stage avascular necrosis (AVN) lesions, which in turn influence management decisions.

We present a male in his early 40s, diagnosed with a right-sided AVNFH (Ficat-Arlet stage I) five years previously at another centre. A number of risk factors were identified, such as chronic alcohol abuse, smoking, obesity, and Klinefelter’s syndrome. A 'watch and wait' approach was adopted, which included advice on reducing risk factors and commencement on aspirin and alendronic acid. However, his pain had recently increased, resulting in a significant reduction in mobility and an increased reliance on opiates. MRI demonstrated progression to Ficat-Arlet stage II, and the appearance of an additional smaller, second lesion located more medially in the same femoral head. Due to his symptom severity, he was offered a THR. In view of his young age, he came to our tertiary referral centre for a second opinion. He elected for a simultaneous dual surgical decompression of both AVN lesions and biological stimulation for bone-guided regeneration. This involved the delivery of growth factor (bone morphogenetic protein), progenitor cells, and a scaffold/matrix. At 36 months post-operatively, he continued to have the full, pain-free weight-bearing functional capacity, with radiographic imaging demonstrating no residual AVN or femoral head structural collapse.

This was a unique case of bi-focal femoral head lesions, treated successfully with decompressions and biological enhancement using the 'diamond concept' for bone repair. In similar situations, when salvage of the femoral head is the preferred treatment option, such a strategy should be considered in the surgeon’s armamentarium.

## Introduction

Avascular necrosis (AVN), or ‘osteonecrosis’, refers to the death of bone cells and tissue, as a result of a compromise to the blood supply of the bone [1]. This leads to the loss of integrity of the subchondral bone structure, resulting in subcortical bone collapse and, subsequently, the development of secondary osteoarthritis [2].

AVN can arise in a number of vulnerable skeletal sites, but most frequently occurs in the hip joint [3]. It is a relatively uncommon phenomenon, affecting between 1.4 and 3.0 individuals per 100,000 in the United Kingdom, with AVN of the femoral head (AVNFH) accounting for the majority of cases (75.9%) [3]. It is more common in males, particularly between the ages of 24 and 44 years and typically affects women at a slightly older age (at 55-75 years) [4].

Patients diagnosed with AVNFH can be divided into two categories based on disease aetiology: trauma-induced AVNFH with no identifiable risk factors, and non-traumatic AVNFH, attributed to a number of underlying risks. Barquet et al. (2014) reported 1.37% of traumatic AVNFH arose directly from trochanteric fractures [5]. However, a number of recognised atraumatic risk factors are also implicated, the most common of which are alcohol excess and corticosteroids. Other aetiological factors that may be summative include male sex, heavy smoking, hyperlipidaemia, a family history of AVN, blood disorders such as sickle cell disease, drugs including chemotherapy, and marrow infiltrating conditions such as Gaucher’s disease [6].

The diagnosis of AVNFH can be challenging, especially in the absence of trauma. The presence of persistent groin and/or upper anterior thigh pain made worse by movement and weight-bearing and arising at night should raise a red flag alongside the aforementioned risk factors [7]. AVNFH may also progress to being bilateral [6]. Interestingly, AVN may sometimes be asymptomatic after being found incidentally on imaging. Clinical examination is non-specific, although can help differentiate an intra-articular pathology (especially pain on internal rotation) from the back and knee.

Plain radiography and magnetic resonance imaging (MRI) are most commonly used to diagnose and stage AVNFH. X-ray is known to lack sensitivity, particularly in early disease, so a low threshold for MRI should be considered. A number of scoring systems have been proposed to stage AVN-related bone abnormalities. The one suggested by Ficat and Arlet is commonly used [8]. At each of the four stages, the patient is presumed symptomatic, and abnormal signal changes and oedema are present on MRI (stage I: normal/mild osteopenia on X-ray; stage II: abnormal X-ray with osteopenia and/or subchondral cysts (pre-collapse); stage III: a crescent sign and evidence of cortical collapse on X-ray; stage IV: secondary osteoarthritis of the joint) [8]. In contrast, the Steinberg classification system provides a semi-quantitative method for evaluating the extent of involvement in relation to the femoral head surface (grade A: <15%; grade B: 15-30%; grade C: >30%) [9]. These grades have been shown to predict the risk of femoral head collapse.

Management of the disease is based on symptom severity and radiological staging. Any secondary causes should be removed where possible, e.g., alcohol and smoking cessation. Femoral head collapse (Ficat-Arlet stage III and IV) will normally progress to a hemiarthroplasty or total hip replacement (THR) depending on patient age; surprisingly, this latter approach to AVNFH management accounts for 5-12% of elective THR procedures annually [6]. There is no consensus regarding treatment of the earlier stages I and II where collapse has not already occurred. Medically, bisphosphonates (oral or intravenously) and vasodilator-prostacyclin analogues (e.g., iloprost) may be initiated, although evidence is equivocal [10]. From a surgical perspective, patients are normally offered a core decompression and/or bone grafting with additional therapies, for example, stem cells and growth and angiogenic factors, as well as an osteotomy [11].

Early identification of AVN before bone collapse is important to consider either non-operative or the ‘least-invasive’ surgical strategies to minimise the risk of progression to a THR. Herein, the case of a patient with unilateral AVNFH with a bi-focal lesion is discussed, focusing on the aetiology of the development of a simultaneous dual lesion, and the treatment modality implemented to optimise the outcome.

## Case presentation

A male patient in his early 40s presented to our tertiary centre for the evaluation of localised and worsening right hip pain and consideration of hip reconstruction. He had been seen five years previously at another hospital when he was given a diagnosis of AVNFH, based on clinical history, physical examination, and radiographic findings. His surgeon did not consider any intervention at this stage as his symptoms were minor, and his lesion was of Ficat-Arlet stage I. A decision was made to treat him non-operatively, and to adopt a 'watch and wait' strategy. Nonetheless, he was commenced on aspirin, and after several months, he was prescribed weekly alendronic acid for mild ongoing symptoms. However, as his symptoms suddenly deteriorated, the patient was referred to our centre for a second opinion. On presentation, he was in significant and debilitating pain, requiring daily morphine for symptom control, which in turn reduced his walking capacity and ability to perform activities of daily living. Physical examination highlighted the presence of substantial pain on internal and external rotation of the right hip, and pain on the straight leg raising past 60 degrees. Both lower limbs were neurovascularly intact throughout.

It was considered that his long history of alcohol excess and smoking were the main predisposing factors to his AVN development. In addition, he was also obese (BMI = 32.1), had Klinefelter’s syndrome, a chromosomal condition for which he was treated with testosterone, and had a background of compromised mental health.

**Figure 1:**
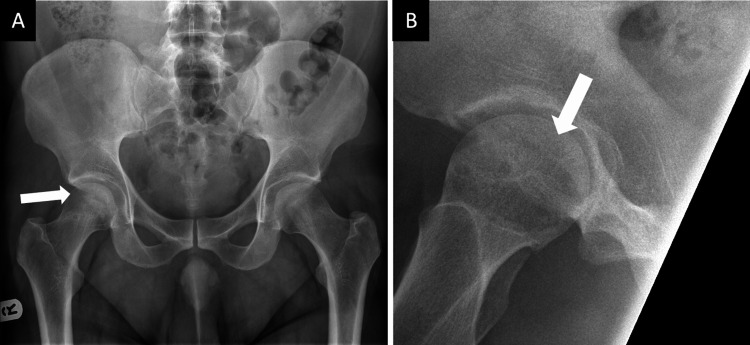
Anteroposterior (A) and lateral (B) pelvic radiographs of the right hip joint demonstrating avascular necrotic lesions (white arrows) in the right femoral head.

**Figure 2:**
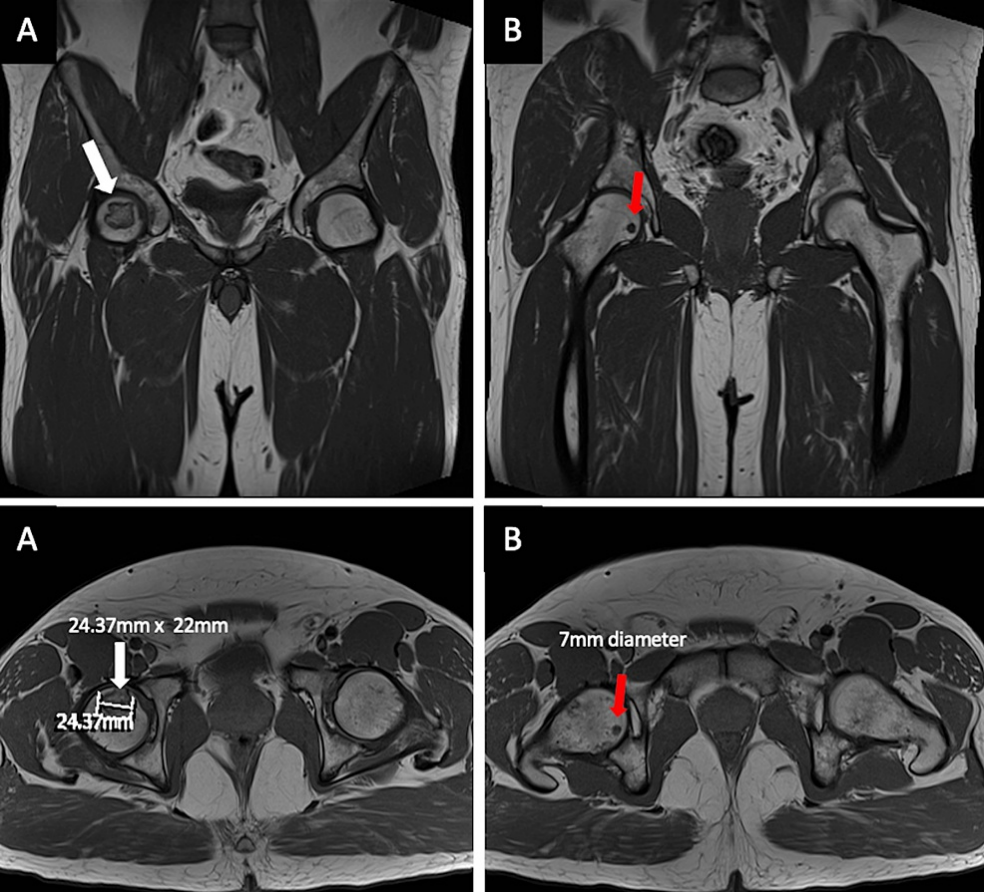
MRI radiographs demonstrating (A) a larger AVN lesion (white arrows), abutting the right anterior superior femoral cortex, measuring 25 x 22 mm, and (B) a smaller AVN lesion (red arrows), located more medially, measuring 7 mm in diameter. The right femoral head remains spherical in shape, with no articular irregularity, collapse, or surrounding tissue degeneration. AVN: avascular necrosis.

New anteroposterior (AP) and lateral pelvic X-rays of the right hip (Figure 1), as well as an MRI scan (Figure 2), were obtained. These investigations confirmed a Ficat-Arlet stage II AVNFH diagnosis. Interestingly, the MRI revealed two AVN lesions: a larger lesion, which abutted the anterior superior femoral cortex measuring 25 x 22 mm, and a smaller one, which was located more medially and measured 7 mm in diameter. It was noted that on MRI, the femoral head remained spherical, with no articular irregularity, collapse, or surrounding tissue degeneration. His blood test results were unremarkable, except for a raised alanine aminotransferase (ALT) secondary to alcohol abuse.

Due to the severity and worsening of his symptoms, both surgical and non-operative management strategies were discussed. The patient was offered two options: either to continue with pain management until the point of disease progression to femoral head articular collapse necessitating a THR or to undergo operative intervention in the form of decompression and delivery of a growth factor and progenitor cells in association with a collagen scaffold/matrix. The patient opted to proceed with the latter operative intervention due to the severity of his symptoms and to have a chance at avoiding a THR, taking into consideration his relatively young age [12].

**Figure 3:**
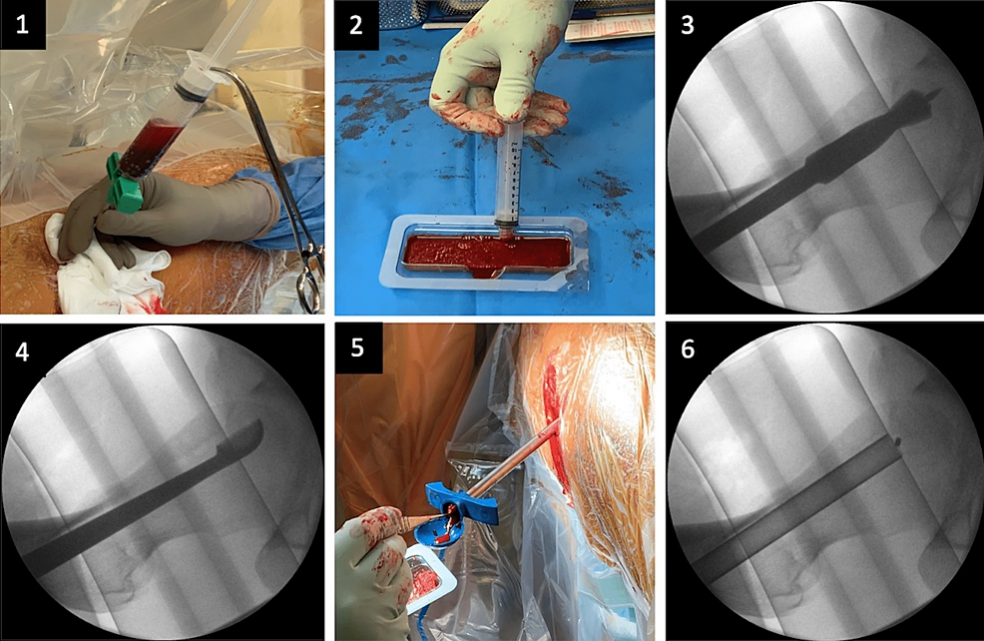
Intra-operative images showing (1) aspiration of bone marrow from right iliac crest; (2) loading of Vitoss foam with BMA and PRP to be used as a scaffold/matrix; (3) two-step reaming process to decompress AVN lesion under image intensifier; (4) debridement of AVN lesion using curettage; (5-6) delivery of loaded Vitoss foam into the area of AVN. AVN: avascular necrosis; BMA: bone marrow aspirate; PRP: platelet-rich plasma.

The operative procedure was as follows (Figure 3). The patient was positioned supine on a fracture table and at induction, received prophylactic antibiotics. The left leg was placed in flexion to allow easy lateral fluoroscopic evaluation for accurate targeting of the AVN lesion. A small stab skin incision was made on the lateral border of the right proximal femur. Under image intensifier (II) control, a guide wire was inserted into the centre of the anterior superior lesion. Using a two-step reaming process (8 mm reaming followed by 12 mm), the centre of the lesion was decompressed and debrided with a curettage. Using the same entry point on the lateral border of the proximal femur, a guide wire was advanced to the centre of the medial femoral head lesion. This lesion was then decompressed with an 8 mm reamer. Subsequently, using a trocar, bone marrow aspirate (BMA) (containing progenitor cells) was aspirated from the right iliac crest (60 ml), which was concentrated to 7 ml using the MarrowStim device (Zimmer Biomet, Warsaw, IN). From the right elbow, 60 ml of peripheral blood was withdrawn, which was also concentrated in 7 ml of platelet-rich plasma (PRP) containing autologous growth factors. Vitoss foam (Stryker, Kalamazoo, MI) was used as a scaffold/matrix and was loaded with both the concentrated BMA and PRP. Using an appropriate delivery tube, the loaded Vitoss foam was delivered within the area of AVN under II control. Subsequently, the entry point on the lateral femoral cortex was sealed with a cancellous Tutobone block (xenograft), to prevent the biological-based therapy delivered at the AVN lesions from escaping to the soft tissue envelope of the right proximal femur. The skin was then sutured with a 3/0 nylon stitch and a wound dressing was applied.

**Figure 4:**
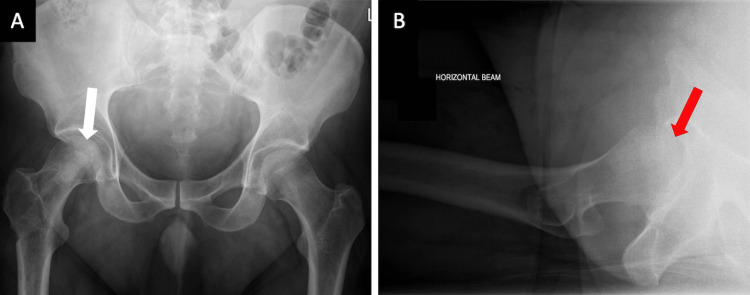
Post-operative anteroposterior (A) and lateral (B) radiographic views of the pelvis and hip joint three years after surgery demonstrating bone modelling. There is no residual AVN or femoral head structural collapse present. AVN: avascular necrosis.

Immediate post-operative care involved thromboprophylaxis (enoxaparin sodium, 4,000 units) treatment for eight weeks and immobilisation (non-weight bearing on the right side), using elbow crutches for three months. Smoking and alcohol cessation was also encouraged. He then progressed to fully weight-bearing without aids and was referred to physiotherapy for strengthening and conditioning of the muscles around the hip. By 12 months, the patient had a full, pain-free weight-bearing functional capacity. At the three-year follow-up appointment, the patient remained pain-free (no intake of painkillers) and maintained full mobility. Final radiographic imaging at this point demonstrated no residual AVN or femoral head structural collapse on both AP and lateral X-rays; bone remodelling was present, and the patient was delighted with the outcome of treatment (Figure 4).

## Discussion

Treatment of AVNFH remains a challenging clinical entity to address. Operative intervention is usually recommended for pre-collapsed stages of the disease and involves core decompression, core decompression with biological-based therapies, and an osteotomy, and in the advanced stages, a THR is recommended [13].

This case raises a number of interesting points. The patient was unusual in that he had mild symptoms and radiological evidence of AVN for five years prior to his presentation at our centre. There was no history of previous physical trauma, although he was noted to have a number of long-standing risk factors. Lafforgue (2006) had previously suggested that AVN often occurs within six months of risk factor exposure [14], but this was not likely to be the case here. His risk factors included longstanding excess alcohol consumption, being obese, and smoking. An additional risk caused by Klinefelter’s syndrome was also possible; this condition is known to be associated with the development of skeletal abnormalities and potentially a prothrombotic state [15]. This patient had been advised to stop drinking and smoking and to reduce his weight, although whether he did this is uncertain.

It is interesting to consider why his symptoms suddenly deteriorated despite being relatively stable for five years. This was also despite being treated with aspirin and bisphosphonates. This demonstrates the unpredictable clinical course of this pathology and the potential risk of progression of the lesion, as seen in this case, by progression to a Ficat-Arlet stage II and extension into two areas of the femoral head. The fact that he had two AVN lesions was unusual, and we have found no similar published records in the literature. The location of the lesions was not atypical, with one being substantially larger than the other. When initially reviewed by our team, he appeared unhappy as his symptoms were significantly affecting his quality of life and he was requiring regular opioid pain relief. This may have influenced his decision to opt for the decompression, rather than continuing to wait for sufficient radiological changes that would warrant a THR.

Noteworthily, the likelihood of AVN lesions progressing and causing future risk of femoral head collapse is dependent on their size and location. Our patient was classified as a Ficat-Arlet stage II and Steinberg grade B, which would give him a more than 85% chance of progression, as described in the literature [16]. Thus, in this respect, the decision to proceed with an operative intervention was justified.

The objective of decompression is to create a channel into the necrotic lesion to relieve intraosseous pressure and to provide a pathway to restore blood flow to the diseased bone [17]. In a meta-analysis of 24 reports analysing 239 patients at stage II (Ficat-Arlet), decompression has been shown to be successful in 65% with respect to femoral head survival [18]. In recent years, a number of adjunct therapies have also been offered, although their true benefit is uncertain. In our case, the patient received a bone matrix, impregnated with PRP and stem cells obtained from the iliac crest, which was applied through the newly-bored bone channels.

It should be borne in mind that decompression procedures are not without risk. It is known that they may increase the risk of femoral neck fracture and result in damage to articular cartilage [19]. This risk may have potentially been increased by two decompressions in our case. It would therefore suggest that the decision to do this double procedure should not be taken on lightly and that the operator is highly experienced. It is also noteworthy that rehabilitation is a lengthy process and still requires regular follow-up appointments to maintain the range of movement and strengthening of the surrounding muscles. In addition, as he was non-weight bearing for three months, this may not suit some more active individuals.

However, the treatment implemented did contribute to a full, pain-free range of right hip movement with no further progression of the disease. We believe that our strategy, following decompression to augment the local avascular environment in the femoral head with cellularity, inductivity, and conductivity, is more powerful in terms of creating a biological stimulus for bone-guided regeneration compared to core decompression alone. The value of this so-called ‘diamond concept’ applied in this case has been well described in the literature in other environments, such as in long bone non-unions and critical-size bone defects [20]. The availability of these three important components of bone healing induced a local osteogenic response in a timely fashion. This is extremely important to prevent articular head collapse after the three-month period of immobilisation when the patient would restart impact-related activities, thus substantially loading the femoral head from a biomechanical perspective.

## Conclusions

In conclusion, femoral head AVN lesions may be quiescent for a long time, but they can become active again with deterioration at any time point, even after five years. Treatment with core decompression with advanced biological-based therapy, as used for our double AVN lesion, could be considered for a successful outcome, as seen in this unique patient.
